# Please Wait, Processing: A Selective Literature Review of the Neurological Understanding of Emotional Processing in ASD and Its Potential Contribution to Neuroeducation

**DOI:** 10.3390/brainsci7110153

**Published:** 2017-11-17

**Authors:** Eric Shyman

**Affiliations:** Child Study, St. Joseph’s College, Patchogue, NY 11772, USA; eshyman@sjcny.edu; Tel.: +1-631-687-1222

**Keywords:** Autism Spectrum Disorder, neuroimaging, emotional processing, neuroeducation

## Abstract

Autism spectrum disorder (ASD) and its corresponding conditions have been investigated from a multitude of perspectives resulting in varying understandings of its origin, its outplay, its prognosis, and potential methods of intervention and education for individuals with the disorder. One area that has contributed significantly to providing a different type of understanding is that of neuroscience, and specifically neuroimaging. This paper will offer a selective literature review of research that investigates the role of emotional processing in ASD, and how a deepening of this line of understanding can be used to inform more comprehensive educational practices.

## 1. Introduction

The ever advancing science of neuroimaging has been responsible for providing new and deeper understandings of a number of conditions over the past several decades. Perhaps one the fields in which its contributions has been most valuable is regarding the often enigmatic condition of autism spectrum disorder (ASD). While research investigating ASD has benefited from the contributions of a large cross section of disciplines including psychology, linguistics, and education, among others, the specific contributions made by neuroimaging has been of particular value as it allows for biophysiological observation and evidence gathering for phenomena that were previously understood only in behavioral or cognitive terms.

Neuroimaging studies, comprised mainly of magnetic resonance imaging (MRI), functional magnetic resonance imaging, blood oxygen level dependent (BOLD) signals, and diffusion tensor imaging (DTI), have contributed to an enhanced understanding of ASD on a global level, adding to the legitimacy of ASD being primarily a condition that is neurological in origin [1,2]. ASD has been investigated neurologically within a number of contexts, with studies focusing on overall brain volume, cerebellar volume, thalamus structure and function, amygdala structure and function, issues in the corpus callosum, the fusiform area, and general neural connectivity [3,4,5].

This paper will delineate current findings from the literature that support the origin of atypical emotional processing in ASD to neurological conditions. In addition to the literature review itself, this paper will further attempt to proffer a practical use for the neurological research in the area of emotional processing by outlining how such an understanding can contribute to advances in neurologically based education, or “neuroeducation”, potentially enhancing both decision making processes and clinical practices. Using a non-random, selective sampling literature review process, multiple articles were chosen that demonstrate findings evidencing the processing differences of both emotions and sensory stimuli in the brain of individuals with ASD. Literature searches using relevant search terms such as “brain”, “emotional processing”, “neuroimaging”, and “autism” (amongst others) were conducted using Psycinfo, Psycarticles, ERIC, OmniFile, and Academic Search Elite through a database provided by a private institution of higher education. The articles were chosen based on a goodness of fit to the topic of social-emotional processing for individuals with ASD (at the author’s discretion), whether the publication was in a peer-reviewed journal no earlier than 2004, and whether the full text was available. This sample does not represent an exhaustive review of the literature. Rather, it is suggested to be a representative exploration of the general findings of the larger research. All articles that were accessed in full-text form by a pre-chosen date were included in the analysis. The following criteria were required for inclusion in the sample:

1. The paper was published in a peer reviewed journal.
2. The paper was published no earlier than 2004.
3. The paper handled an issue of emotional processing in ASD using neuroimaging techniques such as MRI, fMRI, BOLD, and DTI.
4. The paper generated a theoretical framework based on research involving emotional processing, neurological research, and/or neuroimaging (NOTE: These articles were included primarily to theoretically support neuroimaging research in emotional processing and were complementary to the main literature review).

## 2. Emotion, Facial Processing, and Amygdala Activation: The Literature Review

It has long been behaviorally evidenced that individuals with ASD appear to respond differently to sensory stimuli in the environment [6]. While some individuals display hypersensitivity, or higher than typical reactivity to environmental, others display hyposensitivity, or lower than typical reactivity to stimuli. This difference in processing as well as any resulting behavior considered to be symptomatic (such as hand flapping, jumping, vocalizing, or avoidant physical behaviors including ear or eye covering) of autism spectrum disorder is considered as being atypical. Increasingly, neuroimaging is demonstrating clearly that the brain regions involved in the sensory processing of individuals with ASD are both structurally and functionally different than those without [7,8]. Additionally, emerging evidence also suggests that sensory processing atypicality can also be associated with atypical behavior as well as atypical displays of emotion, as demonstrated via the common language of self-stimulatory or stereotypical behavior [9]. One study evidences the prominence of atypical sensory processing among individuals with ASD by investigating 281 children between the age of 3 and 6 years with ASD and an age-matched control group. Each participant was administered the Short Sensory Profile to gauge sensory processing tendencies. Results suggested that 95% of the children with ASD demonstrated some level of sensory dysfunction, with the greatest differences on the “seeks sensation”, “audio filtering”, and “tactile sensitivity” subscales. Additionally, as compared to the control group, participants with ASD performed differently on 92% of the total items (*p* < 0.001) [10].

One common line of research in brain-based sensory differences among individuals with ASD involves visual processing, especially that of emotional facial expressions. A study investigating whether individuals modulate their attention when shown social stimuli via faces and nonsocial stimuli via houses. Sixteen individuals with ASD and 16 age-matched control participants were included in the study. Using mass-univariate and region of interest analyses, the researchers found that while individuals with and without autism showed responses to houses that were modulated by attention, only the individuals without autism demonstrated attentional modulation of face-selective regions. The lack of attentional modulation among individuals with ASD for the social stimuli aligns with the general understanding of atypical responsivity in social situations [11].

Researchers investigated whether facial processing differed for children with ASD in four specific types of situations: (a) social dynamic, or video clips of social interactions (b) social static, or pictures of social interactions, (c) isolated dynamic, or videos of alone individuals, and (d) isolated static, or pictures of alone individuals. Results indicated that, compared to controls, the participants with ASD differed in facial processing only for social dynamic scenes, in which they exhibited decreased fixation for eye regions and increased fixation for body regions. These findings suggest that eye contact as a global issue may not be as significant as the role of eye contact in specific social situations [12].

The existence of differences in emotional processing and expression is also well-established in the literature. Neuroscientists conducted a study with 27 young adults with ASD along with 40 of their relatives, as well as 35 adult controls, in order to investigate the role of cognitive processing and emotion. Using the Toronto Alexithymia Scale and the Beck Depression Inventory, the researchers concluded that the participants with ASD were significantly more impaired emotionally than their non-autistic counterparts [13].

A theoretical perspective investigating amplified emotional responses and poor emotional control specifically, which was conceptualized as compromised emotional regulation. The authors suggested that the compromised emotional regulation among individuals with ASD is likely a multifactorial issue, with some aspects potentially acting in a negatively synergistic fashion. Therefore, a better understanding individual contributors to compromised emotional regulation is likely to result in more effective individualized treatments [14].

Additional researchers contributed to further understanding the emotional situation of individuals with ASD and the brain’s potential role in it by conducting a study using functional magnetic resonance imaging (fMRI). Using 15 male participants with ASD as well as 15 control participants without ASD, researchers gathered data using pictures from the International Affective Picture System on two separate occasions, and compared the participants’ answers with activity measured during the fMRI. Interestingly, results indicated that individuals with ASD differed in brain activity from control participants regardless of whether they self-identified difficulty processing emotions. These findings suggest that difficulties in emotional functioning and regulation may be likely due to gross brain function rather than cognitive or mentalizing functions, establishing the very real possibility of organic difficulties in processing emotions [15].

While the brain is undoubtedly a complex organ ever posing challenges to neuroscientists, there are some aspects of brain research that appear to be relatively consistent. One of these areas is the apparent role of the amygdala in emotional functioning for human beings. The amygdala is an almond-shaped structure that is considered to be part of the brain’s limbic system. Geographically, it is located in the medial temporal lobe, specifically at the anterior end of the hippocampus. Generally speaking, the amygdala is associated with processing emotions, particularly fear and pleasure, and may also be associated with emotional memory.

An early attempt at delineating an amygdala-based theory of ASD was proposed which resulted from fMRI studies of individuals with ASD and their ability to judge emotion based on cues from other people’s eyes. The imaging demonstrated that the participants did not show activation in the amygdala when making such judgments, while participants without ASD did, as was expected [16].

Additional theorists contributed to Baron-Cohen et al. (2000) neurodevelopmental model for an amygdala-based theory of emotional processing for individuals with ASD; one which is directly connected with social cognition. The theory focuses primarily on the reciprocal neurological relationship between the orbitofrontal area of the cortex and the amygdala. Specifically, the orbitofrontal area of the cortex receives far more signals from the amygdala than other areas of the frontal cortex. Because these areas communicate closely, the connection between their various functions (social dynamics and emotional regulation) are likely interconnected [17]. As suggested by the authors:

…the anatomical organization and reciprocal relationship between the amygdala and the orbitofrontal cortex implies that these brain regions may share a close functional relationship within a system essential for the maintenance of intra-specific social bonding and the self-regulation of emotional states.

(p. 102)

This notion becomes important in the study of ASD in that the authors suggested that much of the emotionally based symptomatology of ASD could potentially be explained by issues with the medial temporal lobe, of which the amygdala is a part, thus affecting the functionality of the orbitofrontal-amygdala circuit.

Scientists used magnetic resonance imaging (MRI) to measure both the total cerebral volume and specific amygdala volume of individuals with ASD as compared to those without. Participants included individuals aged 7.5–18.5 years in four diagnostic groups: (a) autism with mental retardation (*n* = 19); (b) autism without mental retardation (*n* = 27); (c) Asperger syndrome (*n* = 25); (d) age-matched individuals without a diagnosis (*n* = 27). Results showed that children with ASD (both with and without MR) had amygdalae that were larger in volume (specifically between the ages of 7.5 and 12.5) than those without. These results suggest that both the occurrence of ASD as well as age may have an effect on the volume of the amygdala [18].

Reseachers investigated the role of the amygdala in emotion and face processing comparing 15 children with high functioning autism with 12 children who were typically developing, aged 8–12 years old. Participants were required to match facial expressions with people while fMRI was conducted. Results indicated that the children with autism, though apparently able to match emotions with faces, showed diminished activation in the fusiform gyrus, an area thought to be associated with facial processing, as well as the amygdala, thought to be associated with emotional processing [19].

A further study investigated 24 participants, 12 with autism and 12 typically developing controls, further investigating the role of attention in emotional processing. Researchers conducted fMRI scans on each participant while they viewed emotional faces, specifically happy, sad and angry, and emotionally neutral faces. While this study did not demonstrate any group difference between attentional bias between emotional and neutral faces, fMRI did indicate greater right amygdala activation among the ASD group as compared to the control group, as well as different connectivity. Additionally, participants with ASD demonstrated stronger connectivity between the amygdala and prefrontal cortex, though weaker connectivity between the amygdala and the temporal lobe [20].

An additional study using fMRIs for 31 adults with ASD and 25 age-, gender-, and IQ-matched controls to investigate brain reactivity, including amygdala action in response to facial expression matching, specifically of fear or anger. The researchers also investigated the connection between brain activity and self-reported social anxiety measured by the Social Avoidance and Distress Scale (Watson & Friend, 1969). Among a number of differences in brain activation between controls and participants with ASD, individuals with ASD showed increased activation in the amygdala, which also correlated with high levels of self-reported social anxiety [21].

An MRI investigation utilizing 52 children with a mean age of 11.9 years, 29 of which had a comorbid diagnosis of an anxiety disorder and 24 of which did not was conducted. An additional control group of 37 age- and intellectually matched children who were deemed as “typically developing” was also included. Controlling for total brain volume, results indicated that the individuals with ASD and anxiety showed decreased right amygdala volume compared with those individuals with ASD but without anxiety as well as with the control group. These results suggest that anxiety may play an additional role in the neurological phenotype amongst individuals with ASD, and may contribute to understanding the role that the amygdala plays in the processing of emotions specifically for individuals with ASD [22].

Researchers investigated the connection between eye contact avoidance, a common reported trait in individuals with ASD, with amygdala activation. Participants included 17 males with ASD as well as 16 controls considered to be neurotypical. Researchers showed an initial image of a fixation cross, toward which participants aimed their eyes, followed by a series of faces to participants and asked them to gauge emotion with either the mouth or the eyes occupying the area of the initial fixation cross. Using eye-gaze measurement technology as well as a blood oxygen level-dependent (BOLD) signal in the amygdala, the researchers were able to detect differences in both amygdala activity and eye gaze. Unsurprisingly, individuals with ASD gazed more often away from than toward the eyes as compared with controls, as well as different BOLD activity between the two groups, suggesting both behavioral and neurological differences in people with ASD as it relates to facial expression processing and corresponding amygdala activity [23].

Neuroscientists investigated the specific process of amygdala habituation, which is the neurological tendency for the amygdala to decrease in responsiveness when presented with repeated stimuli, and is thought to be indicative of typical brain functioning. This process is important in allowing individuals to regulate and maintain appropriate levels of arousal in predictable social stimuli. The lack of amygdala habituation is thought to be associated with higher levels of anxiety. Data collected from fMRIs of 32 children and adolescents and 56 typically developing control participants were analyzed to measure levels of amygdala habituation in response to pictures of faces showing the emotions of happy, sad, fearful, and neutral. In general, results indicated that the participants with ASD showed decreased amygdala habituation as compared to the typically developing controls. Similarly, amygdala habituation correlated with the individual’s score on the Social Responsiveness Scale, with increased severity indicating decreased habituation. These results suggest that faces, specifically repeated faces, are processed differently amongst individuals with ASD, and that the amygdala is likely to play a role in this experiential difference [24].

A study investigating habituation effects and amygdala activation in response to face stimuli displaying varying emotions using an established habituation index. Using fMRI, 22 individuals with ASD and 24 controls without ASD were evaluated for this study. There were two main findings revealed by the data analysis. First, there was a systematic decrement of reaction time among the control participants, though not among the participants with ASD. Secondly, there was a pattern of habituation evident in the amygdala activation of control participants, but such a pattern was absent in the fMRI of autistic participants. This study is particularly contributive to the knowledge base, as it suggests that habituation can be identified both neurologically as well as behaviorally [25].

Building on the idea that oxytocin can enhance amygdala activity, potentially stimulating the ability to feel empathy and process emotion in a way more indicative of typical brains, the researchers sought to test its effect on individuals with ASD in a double-blind, randomized placebo clinical trial. Administering either oxytocin via intranasal spray or a placebo to 16 males aged 12–19 years old, the researchers found that individuals who received oxytocin as compared to the placebo showed an improvement in the Reading the Mind Through the Eyes Task. This study suggests that understanding the neurological makeup of individuals with ASD can enhance basic understanding of the condition, but can also inform potential treatment and intervention options as well [26]. It is important to note, however, that a further study by the same researchers revealed that oxytocin treatment as compared to the placebo did not demonstrate a benefit among individuals with ASD, leaving the treatment’s effectiveness as yet unresolved [27].

An additional medically based pilot study including 19 adults with ASD in order to investigate the utility of intranasal oxytocin as a treatment for social functioning and repetitive behaviors was conducted. Social functioning was measured using the Diagnostic Analysis of Nonverbal Accuracy and Reading-the-Mind-in-the-Eyes test, among other secondary measures, and repetitive behaviors were assessed by the Repetitive Behavior Scale-Revised. Results indicated statistically significant changes in the social cognition realm (*p* < 0.05) as measured by the Reading-the-Mind-in-the-Eyes test. The authors suggested that the results indicated promise for the potential therapeutic use of oxytocin, but emphasized that more robust studies were warranted [28].

## 3. Conclusions

While this selective literature review only included a sample of the extant research investigating the role of emotional processing amongst individuals with ASD, there are clearly two distinct trends present. First, a difference in emotional processing at the neurological level has been well-established in individuals with ASD. While this tendency has been suggested based on behavioral observations, the evidencing of structural and functional differences in brains of individuals with ASD can allow for a deeper understanding of why these differences exist. Second, while other areas of the brain are likely also associated with differences in emotional processing, the amygdala appears to be one of the central neurological structures responsible for these differences. Increased understanding of this area of the brain may help provide guidance for both biological as well as educational interventions for individuals with ASD that specifically target emotional responsivity.

## 4. Connecting Neurological Understanding of Emotional Processing with Neuroeducation

While enhancing the basic understanding of particular human conditions is valuable in and of itself, such knowledge becomes ever more important when it can be used to enhance the social state of a culture and increase the well-being for those individuals who are a part of it. The emerging field of neuroeducation provides a sound example of how advances in neuroimaging, though scientific in basis, can have a distinct impact on the quality of education provided for individuals with ASD and its more global effect on their ability to socially function in a meaningful way:

Neuro-Education is a nascent discipline that seeks to blend the collective fields of neuroscience, psychology, cognitive science, and education to create a better understanding of how we learn and how this information can be used to create more effective teaching methods, curricula, and educational policy. Though still in its infancy as a research discipline (neuroeducation) is already opening critical new dialogs between teachers, administrators, parents, and brain scientists.

[29]

Adding further to the understanding of how neuroeducation may continue to grow and contribute to the larger body of curriculum and instruction, neuroeducation theorists suggested that the goals of neuroeducation can only be met if five key groups of educational stakeholders participate in its facilitation: (a) practicing teachers; (b) neuroscience researchers; (c) publishers and the popular media; (d) educational policymakers; (e) university-level teacher educators [30].

The potential link between neurological research and educational practice is also highlighted in a series of questions guiding neuroeducation development: (a) Which principles, mechanisms, and theories studied in educational research can be extended on the basis of findings from cognitive neuroscience? (b) Which principles, mechanisms, and theories studied in cognitive neuroscience might have implications for educational research? (c) What research questions can be developed on the basis of these implications? and (d) What form could an interdisciplinary research program take? [31].

As research methodologies and technology in neuroimaging continue to advance, it becomes even more imperative that such research projects be conducted in more practical and applied settings rather than laboratory environments. Because neuroimaging can detect changes in as short a time as a few milliseconds, well before raw behavioral observations can be facilitated, factors that affect the behavior, and potential modulations in that behavior, may be better identified, further enhancing the quickness and efficiency that core issues of individuals may be addressed [3,32].

Specifically regarding the education of individuals with ASD, the benefit of using results from neuroimaging research would be widely applicable. As suggested:

Research on autism is increasingly multidisciplinary. However, there are currently few models of autism that bridge observations between the biological, environmental, and behavioral sciences.

[33]

This unilateral vantage is problematic as there is no single methodology or framework for education that could possibly address the heterogeneous, ever variable, and often unpredictable needs that many individuals with ASD present. Despite the variety of disciplines that contribute to the research in understanding the ASD as a condition, the interventional and therapeutic research appears to emphasize behaviorally based approaches (known also as Applied Behavior Analysis, or ABA) disproportionately to other types of approaches.

Two examples from the USA can be found in the policy of Tricare, the health insurance system of the US Department of Defense, as well as certain policies evident in the Medicaid process, a qualification-based system of subsidized health care available in the US. In the case of Tricare, recent policy implementation has attempted to restrict the provision of “habilitative care” to services based in Applied Behavior Analysis, thus eliminating the possibility for other effective approaches, such as those based on social, emotional, communication principles to be covered. Additionally, some states have been granted waivers by Medicaid allowing ABA providers to be the “sole gatekeepers” regarding interventional decision-making on behalf of families and children with ASD [34]. These practices have garnered enough interest to prompt the United States Department of Education [35] to distribute a memo indicating that

…some IDEA programs may [include] applied behavior analysis (ABA) therapists without including, or considering input from, speech and language pathologists and other professionals who provide different types of specific therapies that may be appropriate for children with ASD.

It is possible that policy decisions that result in the exclusion of other evidence-based approaches occur as a result of a lack of understanding by policymakers on the broader conceptualization of an evidence basis. Advances in neuroscience, especially neuroimaging in particular, can be used to evidence the need to include socio-emotional and socio-developmental approaches in the educational programming for children with ASD, much of which has already established an evidence basis in the literature [36,37,38,39].

In one area, the effectiveness of allowing a combination of both behaviorally based and socio-developmentally based approaches has been showing promise. An emerging methodological practice known as Naturalistic Developmental Behavioral Intervention (NDBI), of which there are a number of examples, has been found to be effective for a variety of issues experienced by individuals with ASD [40].

While the entertainment of such an idea has come only recently in the educational, behavioral, and applied psychology field of research in ASD, had the findings of neuroscience regarding neural structure and processing differences enjoyed a more interdisciplinary attention, this type of progress may have been made sooner and with less resistance. As it is clear that the structural and functional makeup of the brains of individuals with ASD is not only different from those without ASD, but also similar to one another, using these advances to design educational protocols in areas that have been difficult to capture behaviorally (such as socio-emotional approaches) can provide an innovative and sound means to enhancing the current state of education for individuals with ASD.
